# Radiological and Clinical Effects of Exercise Intervention Therapy on Adolescent Idiopathic Scoliosis: A Meta-analysis

**DOI:** 10.1007/s43465-026-01861-8

**Published:** 2026-06-19

**Authors:** Yuxin Zhang, Jiulong Song, Yuxin Fang, Jiexin Gu, Jian Fu

**Affiliations:** College of Physical Education, Yangzhou University, Yangzhou, 225127 Jiangsu China

**Keywords:** Adolescents, Idiopathic scoliosis, Exercise intervention therapy, Meta-analysis, Radiological and clinical efficacy

## Abstract

**Background:**

To evaluate the radiological and clinical efficacy of exercise intervention therapy in improving Cobb angle, axial trunk rotation (ATR), axial vertebral rotation (AVR), and quality of life in adolescents with adolescent idiopathic scoliosis (AIS).

**Methods:**

Literature was retrieved from databases including CNKI, Wanfang, VIP, Web of Science, and PubMed, and screened according to inclusion criteria. Methodological quality was assessed using Cochrane Risk of Bias Tool. This is a meta-analysis based on a systematic review. RevMan 5.4 software was employed to calculate pooled effect sizes for Cobb angle, ATR, AVR, and the Scoliosis Research Society-22 (SRS-22) quality of life questionnaire. This systematic review was registered at the International Prospective Register of Systematic Reviews.

**Results:**

Ten randomized controlled trials (RCTs) were included. Analysis revealed that compared with conventional interventions, exercise therapy significantly reduced Cobb angle [Standardized Mean Difference (SMD) =  −  1.80, 95% Confidence Interval (CI) (− 2.61, − 0.98), *P* < 0.05], significantly reduced ATR [SMD = − 1.55, 95% CI (− 2.31, − 0.80), *P* < 0.05], and significantly enhanced quality of life [SMD = 1.35, 95% CI(0.72,1.98), *P* < 0.05]. However, the pooled results for improving AVR did not show statistical significance [SMD = − 0.09, 95% CI(− 1.14, 0.96), *P* > 0.05].

**Conclusion:**

Exercise intervention therapy significantly improves radiological parameters including Cobb angle and ATR, effectively improves physical function in AIS patients, and also significantly enhances their quality of life.

## Introduction

The primary characteristic of adolescent idiopathic scoliosis (AIS), a three-dimensional spinal deformity of unclear etiology, is a Cobb angle greater than 10° in the coronal plane, which causes the spine to curve laterally [1]. Adolescent idiopathic scoliosis (AIS) is a condition that cannot be disregarded among teenagers, as epidemiological data show that its occurrence ranges from 0.35 to 5.2%, with a significantly higher proportion of female patients than male patients [2, 3]. Although it is generally acknowledged that AIS is directly linked to muscular strength imbalance on both sides of the spine, decreased trunk stability, and aberrant neurological control, the pathophysiology of the condition is still not fully understood [4]. As the third major health killer threatening the health of Chinese adolescents after obesity and myopia [5], AIS not only affects patients' physical appearance and motor function but may also lead to reduced quality of life, mental health issues, and, in severe cases, diminished cardiopulmonary function [6].

The Scoliosis Research Society (SRS) mainly classifies the treatment approaches for growth-related specific scoliosis into two major categories: surgical treatment and non-surgical conservative treatment [7]. However, surgical treatment is associated with significant trauma, high costs, and is primarily suitable for severe cases. It may be overly aggressive for patients with mild to moderate scoliosis.

Non-surgical conservative treatments mainly include brace therapy, pharmacological treatment, and exercise intervention therapy, which are complementary with clear clinical indications [8–10]. Exercise intervention therapy implements early intervention through scientifically designed systematic exercise programs to control scoliosis progression and improve spinal function. It covers all types of scoliosis, including mild, moderate, and severe cases, and constitutes a core component of the non-surgical treatment system [11]. Studies have found that exercise intervention therapy offers potential benefits in improving the Cobb angle, angle of trunk rotation (ATR), and quality of life in patients with adolescent idiopathic scoliosis (AIS) [12]. AIS patients who underwent specific exercise therapy (PSSE) training supported by a tele-rehabilitation system demonstrated significantly greater improvements in Cobb angle compared to those receiving traditional rehabilitation treatment models, along with higher patient compliance [13]. Furthermore, exercise intervention therapy boasts "five major advantages": proven efficacy, ease of operation, high patient compliance, no significant side effects, and low cost. These advantages make exercise intervention therapy particularly suitable for patients with mild to moderate AIS, providing them with a gentler and more effective treatment option.

Exercise intervention therapy, as a conservative strategy targeting neuromuscular imbalance and spinal biomechanics, plays a key role in correcting three‑dimensional spinal deformity and improving clinical and radiological outcomes in AIS. Although exercise intervention therapy is widely applied in the prevention and treatment of adolescent idiopathic scoliosis (AIS) and has garnered policy support, there remains a dearth of studies in the existing literature that substantiate the effect size of exercise intervention therapy in effectively reducing the Cobb angle, angle of trunk rotation (ATR), axial vertebral rotation (AVR), and improving the quality of life (as measured by the Scoliosis Research Society-22 (SRS-22) score) in adolescent AIS patients. Therefore, this study aims to integrate existing research through systematic review and meta-analysis to compare the efficacy differences between exercise intervention therapy and conventional interventions, thereby providing a reference for the development of precise, evidence-based exercise intervention therapy protocols in clinical practice.

## Materials and Methods

This study strictly adheres to the Preferred Reporting Items for Systematic Reviews and Meta-Analyses (PRISMA) guidelines [14]. Furthermore, the detailed methodological protocol for this meta-analysis has been registered in PROSPERO under the registration number CRD420251124169.

### Literature Search

During the literature search process, two researchers were jointly involved. These two searchers conducted the retrieval work independently in a double-blind manner. For Chinese literature, searches were carried out using China National Knowledge Infrastructure (CNKI), Wanfang Data, and VIP databases. For English literature, Web of Science and PubMed were used as the retrieval databases. The Chinese search terms were combined as "idiopathic scoliosis", "scoliosis", "adolescent", "sports intervention", and "exercise training" for the search. The English search strategy employed a combination of Medical Subject Headings (MeSH) and free-text terms. The English search formula was: ("Adolescent Scoliosis" OR "Adolescent Idiopathic Scoliosis") AND ("Sports intervention" OR "Exercise intervention" OR "Physical activity intervention" OR "Physical exercise" OR "Sport-based intervention" OR "Exercise program" OR "Sport program" OR "Physical training" OR "Schroth" OR "SEAS"). The search time frame spanned from January 2015 to July 2025, and a total of 1096 articles were retrieved.

### Research Selection

Inclusion criteria: (1) randomized controlled trials (RCTs), regardless of blinding status; (2) participants aged 8–18 years, without gender restrictions, diagnosed with adolescent idiopathic scoliosis (AIS); (3) the experimental group received exercise intervention therapy, such as Schroth exercise therapy, core strength training and core stability training, with detailed descriptions of  the specific content, frequency, intensity, and duration of the exercise intervention, while the control group received conventional interventions such as observation, health education, or other non-exercise interventions; (4) primary outcome measures included Cobb angle, axial trunk rotation (ATR), axial vertebral rotation (AVR), and Scoliosis Research Society-22 (SRS-22) patient questionnaire scores, with no restrictions on measurement methods.

Exclusion criteria: (1) duplicate publications retrieved from various databases; (2) publications not published in Chinese or English; (3) review articles, case reports, conference abstracts, and publications where original data could not be obtained; (4) single-case report publications.

#### Literature Screening and Data Extraction

Two researchers (Yuxin Zhang and Yuxin Fang) independently conducted literature screening and data extraction. First, duplicate records were removed using EndNote reference management software. Subsequently, both researchers performed an initial screening based on article titles and abstracts. Full-text articles of selected studies were downloaded and reviewed, followed by a secondary screening strictly adhering to exclusion criteria until the final included studies were determined. Data extraction primarily encompassed: study background information (first author, publication year, study region, etc.); subject characteristics (sample size, age, gender, scoliosis curvature type and degree); intervention details (specific exercise intervention methods, frequency, intensity, duration, and control group treatment); and outcome measure data (Cobb angle, ATR, AVR, and SRS-22 score). Disagreements between researchers during literature screening or data extraction were resolved through discussion or consultation with a third researcher (Jiexin Gu).

### Types of Exercise Intervention and Mean Age at Initiation

The included exercise interventions covered Schroth exercise, SEAS, BSPTS, core stability training, core muscle strength training, traction-assisted exercise therapy, dance body training, and specific scoliosis exercises. The mean age at initiation of exercise intervention was 12.6 years (range: 8–18 years).

### Assessment of Risk of Bias in Included Studies

The Cochrane Risk of Bias Assessment Tool [15] was used to evaluate the quality of included randomized controlled trials (RCTs). The assessment encompassed seven domains: random sequence generation, allocation concealment, blinding of participants and investigators, blinding of outcome assessment, reporting of incomplete data, selective reporting of outcomes, and other sources of bias risk. Each domain was rated as “low risk,” “unclear risk,” or “high risk” of bias. Two researchers independently conducted the assessments and cross-checked results.

### Handling of Confounding Factors

Several included studies adopted a combined intervention of exercise and brace. To reduce the confounding effect of brace use, subgroup analysis was performed to distinguish studies with exercise alone versus exercise plus brace, and sensitivity analysis was conducted to verify the robustness of pooled effect sizes. The results remained consistent after excluding studies with combined brace application, confirming the independent radiological and clinical efficacy of exercise intervention.

### Statistical Analysis

Data statistical analysis was performed using Revman 5.4 software. Continuous outcome measures were expressed as standardized mean differences (SMD) with 95% confidence intervals (CI). The *I*^2^ statistic was used to assess heterogeneity among included studies: *I*^2^ ≤ 40% indicated low heterogeneity; 40% < *I*^2^ < 70% indicated moderate heterogeneity; *I*^2^ ≥ 70% signified high heterogeneity [16]. For effect size pooling, the fixed-effect model was used when studies demonstrated good homogeneity; the random-effects model was employed when heterogeneity was significant (meta-analysis heterogeneity test: *P* ≤ 0.10, *I*^2^ > 50%) [17]. Sensitivity analysis was conducted to explore potential factors influencing heterogeneity. Stata 18.0 software was used for sensitivity analysis, sequentially excluding each study to assess the stability of the overall results. Subgroup analysis further examined the effects of Cobb angle, intervention cycle, and intervention therapy on AIS. Finally, all included studies were assessed using the Cochrane Risk of Bias Assessment Manual to identify other potential biases. For the ten included studies, Cochrane risk of bias assessment plots were generated using Review Manager 5.4 software. Funnel plots were employed to evaluate publication bias, with a significance level of *α* = 0.05.

## Results

### Literature Screening Process and Outcomes

**Fig. 1:**
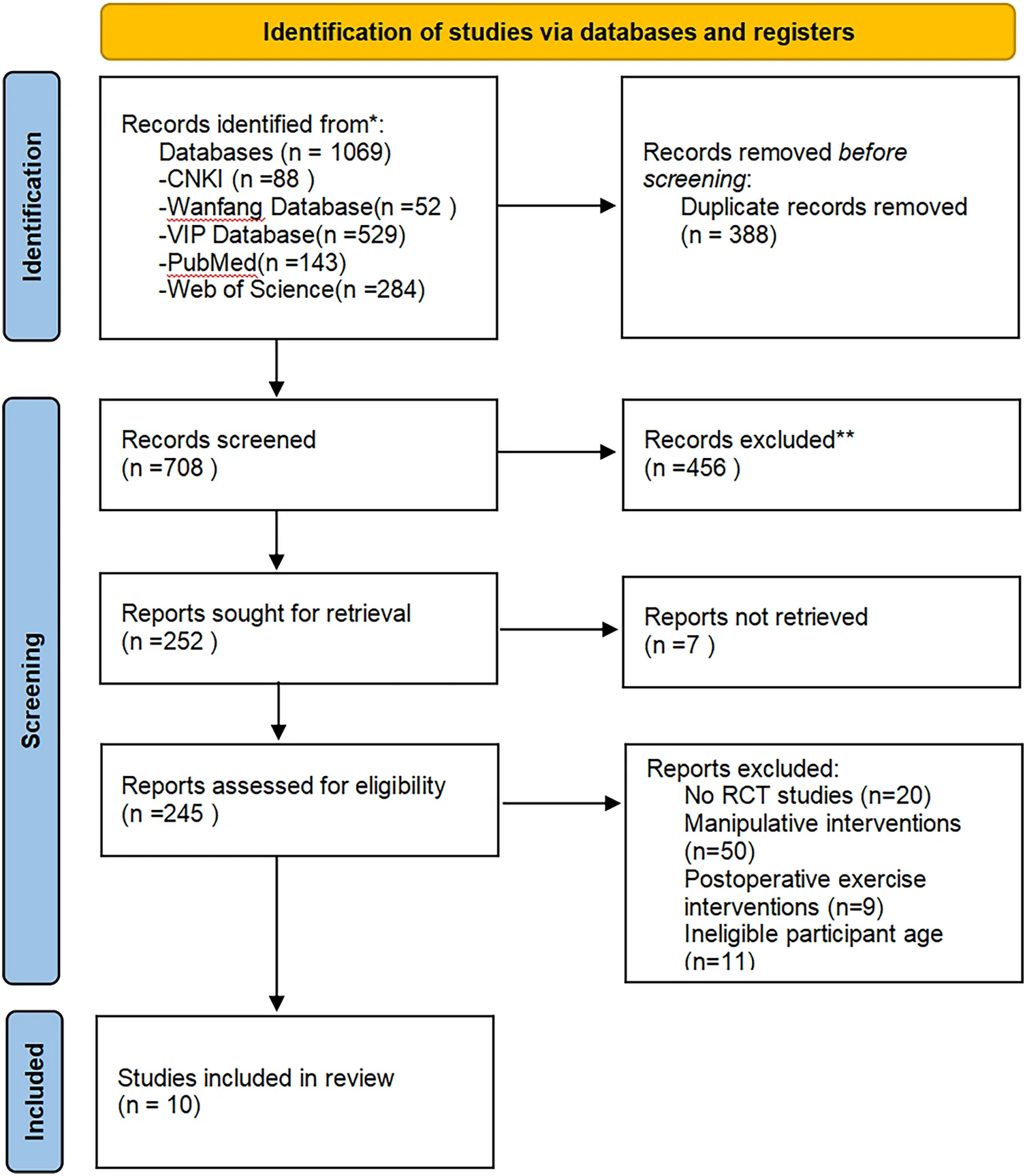
Specific process and results of literature screening

This study identified a total of 1069 relevant publications. After the initial screening, 388 duplicate publications were excluded, leaving 681. Subsequent tiered screening ultimately resulted in the inclusion of ten RCTs. The literature screening process and outcomes are illustrated in Fig. 1.

### Basic Characteristics of Included Studies and Risk of Bias Assessment Results

This study included ten randomized controlled trials involving adolescents aged 8–18 years with idiopathic scoliosis, comprising a total sample size of 498 participants (266 in the intervention group and 232 in the control group). The interventions consisted of various types of exercise therapy; control group interventions included no exercise, standard care, bracing, health education, or routine health promotion. The intervention duration ranged from a minimum of 12 weeks to a maximum of 1 year. Among primary outcome measures, Cobb angle was reported in ten studies, ATR in six studies, AVR in three studies, and SRS-22scores in five studies. Specific sample sizes, ages, interventions, and outcome measures for each study are detailed in Table 1.

**Table 1:** Basic characteristics of included studies

Included studies	Sample size (*n*)		Age (years)	Intervention		Intervention period	Outcome measures
	Experimental/Control	Male/Female	Intervention/Control	Intervention group	Control group		
Arash et al., 2024 [18]	15/10	25/0	16.27 ± 1.44/15.4 ± 1.51	Schroth	No Exercise	12 weeks	①②④
Yagci et al., 2024 [19]	21/11	5/27	13.0 ± 2.15/13.73 ± 1.90	SEAS	Standard care	1 year	①②④
Ji et al., 2024 [20]	45/45	55/35	12.02 ± 1.20/12.16 ± 1.28	Exercise therapy under traction	Brace fixation	3 months	①②③
Liu et al., 2023 [21]	47/34	23/58	12.25 ± 1.78/12.14 ± 1.30	Exercise therapy	Health education	10 months	①②
Liao 2022 [22]	15/15	—	11.2 ± 0.97/11.13 ± 1.02	Core stability training	No exercise	12 weeks	①
Hu 2022 [23]	23/22	11/34	13.91 ± 1.75/13.50 ± 1.97	BSPTS	Daily life health management	6 months	①②④
Guo 2021 [24]	15/15	13/17	11.87 ± 1.46/12.13 ± 2.19	Dance body training	Routine health promotion	20 weeks	①
Zeng et al., 2020 [25]	40/40	36/44	14.9 ± 2.0/14.3 ± 2.1	Core muscle strength training	Brace fixation	90 days	①
Liu et al., 2019 [26]	25/30	15/40	13.2 ± 1.9/13.6 ± 2.0	Exercise training	Orthosis	6 months	①③④
Zhou et al., 2016 [27]	20/10	6/24	11.8 ± 1.8/12.5 ± 2.0	Specific scoliosis exercises	Routine health promotion	12 weeks	①②③④

BSPTS refers to the Barcelona School of Physical Therapy Scoliosis Method; SEAS refers to the scientific exercise approach to scoliosis; "-" indicates not reported; "①" denotes the Cobb angle; "②" denotes the trunk rotation angle (ATR); "③" denotes the apical vertebral rotation (AVR); "④" denotes the Scoliosis Research Society-22 (SRS-22) score

**Fig. 2:**
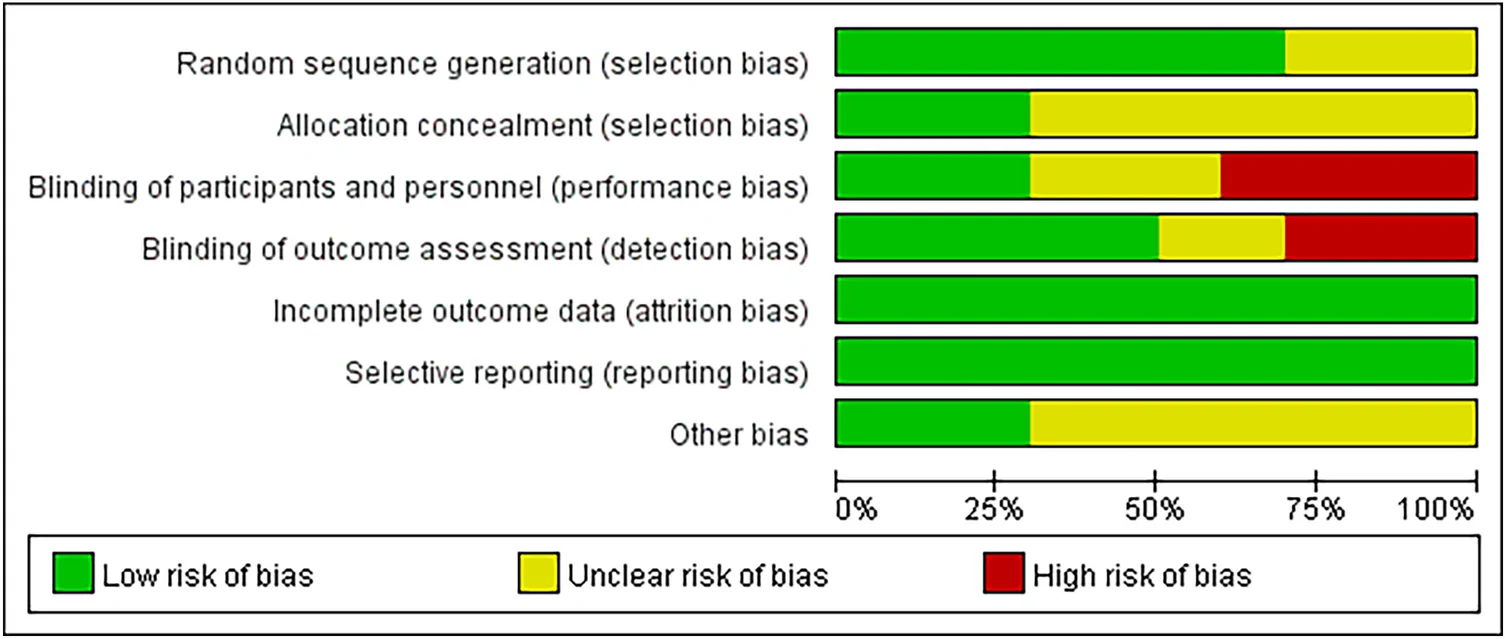
Risk of bias graph. Green markers indicate low risk, yellow markers indicate unclear risk, and red markers indicate high risk

**Fig. 3:**
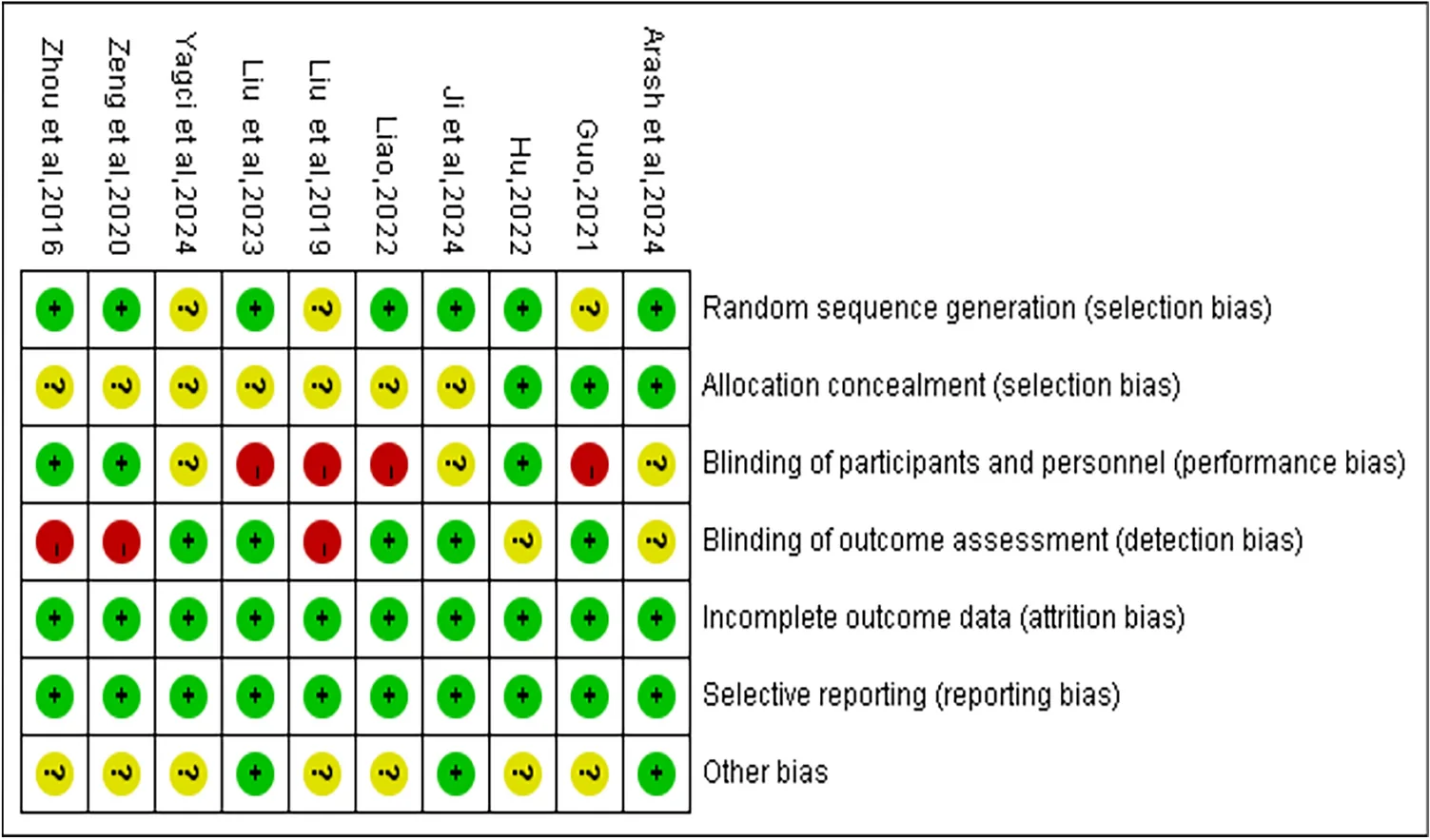
Risk of bias summary

All included studies underwent methodological quality assessment according to the Cochrane Risk of Bias Assessment Manual [15], evaluating the following six aspects: (1) random sequence generation methods: random number tables, etc.; (2) allocation concealment mechanisms: centralized randomization, etc.; (3) blinding design and implementation: whether subjects and implementers were blinded; (4) handling of incomplete outcome data: whether intention-to-treat analysis was applied; (5) selective reporting of outcomes: presence of selective reporting; (6) other potential sources of bias, as shown in Table 2 and Figs. 2 and 3.

**Table 2:** Risk of bias assessment results for included studies

Included studies	Randomization method	Blinding	Allocation concealment	Integrity of outcome data	Selective outcome reporting	Other sources of bias
Arash et al., 2024 [18]	Computer-generated	Not specified	Sealed envelopes	No dropouts	No	Not specified
Yagci et al., 2024 [19]	Not specified	Not specified	Not specified	No dropouts	No	Not specified
Ji et al. 2024 [20]	Grouped by treatment time	Not specified	Not specified	No dropouts	No	Not specified
Liu et al., 2023 [21]	Coin toss	Not specified	Not specified	No dropouts	No	Not specified
Liao 2022 [22]	Random number table	Not specified	Not specified	No dropouts	No	Not specified
Hu 2022 [23]	Computer-generated	Single-blind	Sealed envelopes	No dropouts	No	Not specified
Guo 2021 [24]	Not specified	Not specified	Not specified	No dropouts	No	Not specified
Zeng et al., 2020 [25]	Random number table	Not specified	Not specified	No dropouts	No	Not specified
Liu et al., 2019 [26]	Not specified	Not specified	Not specified	No dropouts	No	Not specified
Zhou et al., 2016 [27]	Random number table	Not specified	Not specified	No dropouts	No	Not specified

Risk of bias was assessed using Cochrane Collaboration criteria. "Not specified" denotes that a study provided insufficient details to judge a domain's risk of bias. "No dropouts" indicates all participants completed the study, resulting in a low-risk judgment for outcome data integrity. All assessments were based solely on information reported in the original publications

### Meta-analysis Results

#### Effects of Exercise Intervention on Cobb Angle in AIS Patients

**Fig. 4:**
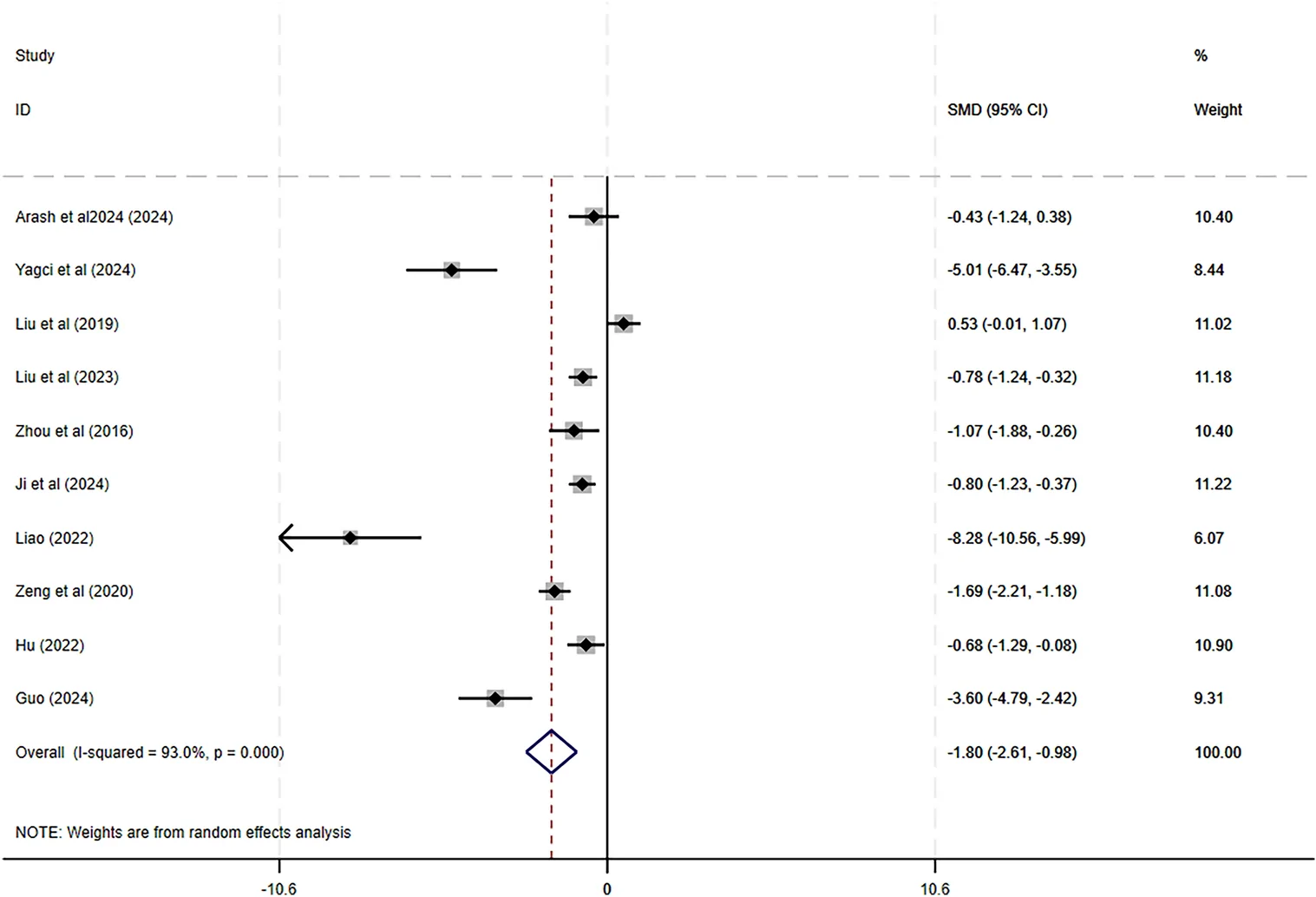
Meta-analysis of exercise interventions improving Cobb angle in AIS patients. Horizontal axis (X-axis): standardized mean difference (SMD) with 95% confidence interval (95% CI). A value left of 0 means exercise intervention reduces Cobb angle vs. control; right of 0 means an increase. Black square: effect size (SMD) of each study, with square size corresponding to its weight in the meta-analysis (see “% Weight” column). Horizontal line (whisker): 95% CI of each study’s effect size. Diamond at the bottom: pooled SMD of all studies, with its width representing the pooled 95% CI. *I*^2^ and *p*-value: heterogeneity indices. Here, *I*^2^ = 93.0%, *p* = 0.000, indicating high heterogeneity; thus, a random-effects model was used. “NOTE”: Weights of studies are calculated via the random-effects model

This study included ten publications using Cobb angle as the primary outcome measure, involving a total of 498 participants: 266 in the intervention group and 232 in the control group. Meta-analysis results are presented in Fig. 4. All ten studies exhibited high heterogeneity (*P* < 0.00001, *I*^2^ = 93%). Compared with the control group, exercise intervention therapy significantly reduced the Cobb angle in AIS patients [SMD = − 1.80, 95% CI (− 2.61, − 0.98), *P* < 0.001].

#### Effects of Exercise Intervention Therapy on ATR in AIS Patients

**Fig. 5:**
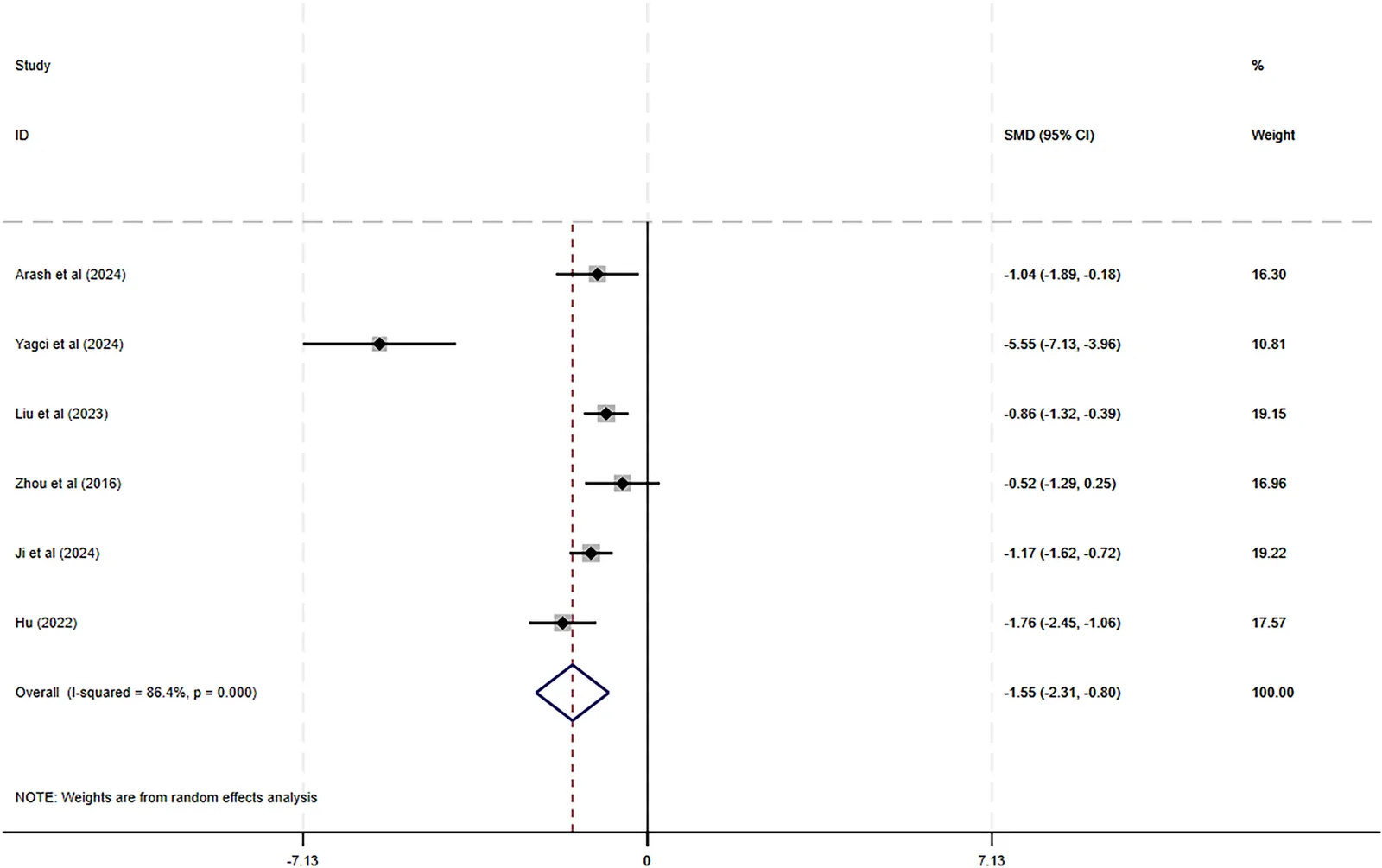
Meta-analysis of exercise interventions improving ATR in AIS patients. Horizontal axis (X-axis): standardized mean difference (SMD) with 95% confidence interval (95% CI). A value to the left of 0 indicates that exercise intervention therapy reduces the trunk rotation angle (ATR) compared to the control group; a value to the right indicates an increase. Black square: the effect size (SMD) of each individual study, where the square’s size corresponds to the study’s weight in the meta-analysis (shown in the “% Weight” column). Horizontal line (whisker): 95% CI of the effect size for each individual study. Diamond at the bottom: pooled effect size (SMD) of all included studies, with its width representing the 95% CI of the pooled effect. *I*^2^ and *p*-value: indices of heterogeneity. Here, *I*^2^ = 86.4%, *p* = 0.000, indicating high heterogeneity among studies, so a random-effects model was applied for analysis

This study included six publications that used ATR as an outcome measure to evaluate AIS patients, involving a total of 303 subjects: 171 in the intervention group and 132 in the control group. The meta-analysis results are shown in Fig. 5. These six studies exhibited high heterogeneity (*P* < 0.00001, *I*^2^ = 86.4%). Compared with the control group, exercise intervention therapy significantly reduced ATR in AIS patients [SMD = − 1.55, 95% CI (− 2.31, − 0.80), *P* < 0.001].

#### Effects of Exercise Intervention Therapy on AVR in AIS Patients

**Fig. 6:**
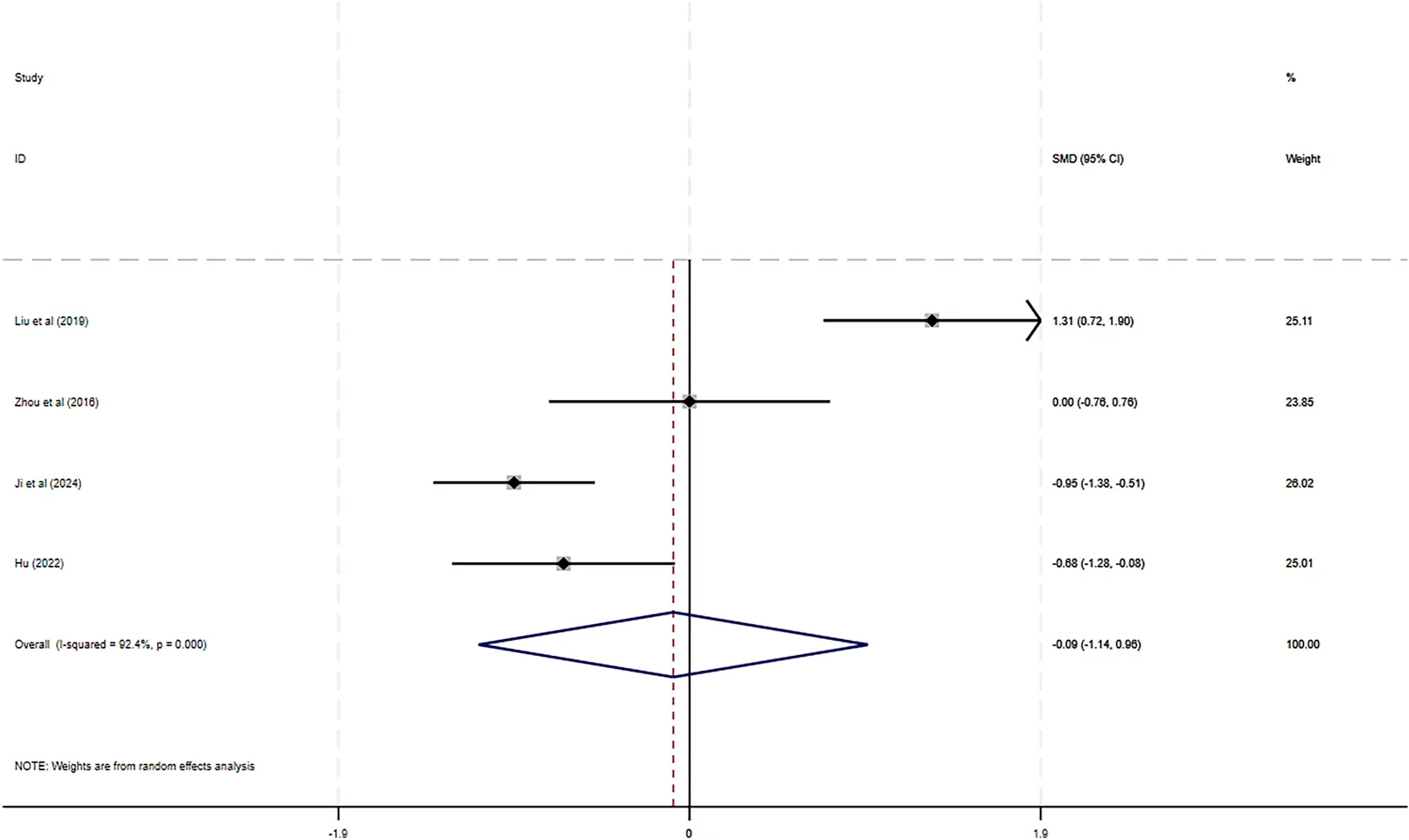
Meta-analysis of exercise intervention improving AVR in AIS Patients. Horizontal axis (X-axis): standardized mean difference (SMD) with 95% confidence interval (95% CI). A value to the right of 0 indicates that exercise intervention therapy improves the outcome (e.g., quality of life or functional score) compared to the control group; a value to the left indicates a decrease. Black square: The effect size (SMD) of each individual study, with the square’s size corresponding to the study’s weight in the meta-analysis (shown in the “% Weight” column). Horizontal line (whisker): 95% CI of the effect size for each individual study. Diamond at the bottom: pooled effect size (SMD) of all included studies, with its width representing the 95% CI of the pooled effect. *I*^2^ and *p*-value: indices of heterogeneity. Here, *I*^2^ = 92.4%, *p* = 0.000, indicating high heterogeneity among studies, so a random-effects model was used for analysis

This study included four publications that used AVR as an effect measure to evaluate AIS patients, involving a total of 220 subjects: 113 in the experimental group and 107 in the control group. The meta-analysis results are shown in Fig. 6. Compared with the control group, exercise intervention therapy reduced AVR in AIS patients. However, since only three studies included AVR as an outcome measure, the pooled results for AVR did not show statistical significance [SMD = − 0.09, 95% CI (− 1.14, 0.96), *P* < 0.001].

#### Effects of Exercise Intervention Therapy on Quality of Life in AIS Patients

**Fig. 7:**
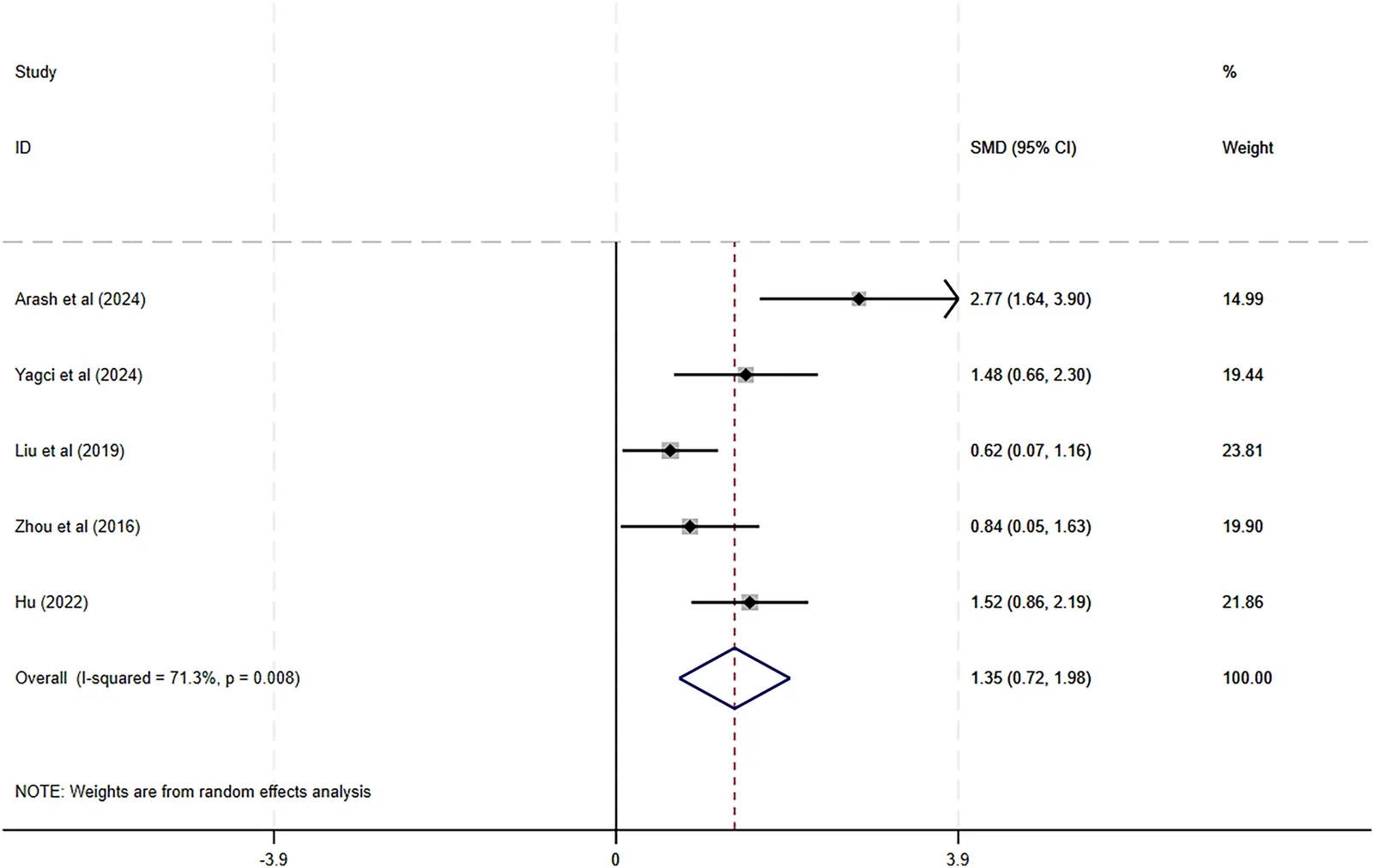
Meta-analysis of exercise interventions improving quality of life in AIS patients. Horizontal axis (X-axis): standardized mean difference (SMD) with 95% confidence interval (95% CI). A value to the right of 0 indicates that exercise intervention therapy improves the outcome (e.g., quality of life or functional score) compared to the control group; a value to the left indicates a decrease. Black square: the effect size (SMD) of each individual study, with the square’s size corresponding to the study’s weight in the meta-analysis (shown in the “% Weight” column). Horizontal line (whisker): 95% CI of the effect size for each individual study. Diamond at the bottom: Pooled effect size (SMD) of all included studies, with its width representing the 95% CI of the pooled effect. *I*^2^ and *p*-value: Indices of heterogeneity. Here, *I*^2^ = 71.3%, *p* = 0.003, indicating high heterogeneity among studies, so a random-effects model was used for analysis

This study included five publications that assessed the quality of life of AIS patients using the SRS-22 patient questionnaire. The total number of subjects was 187, comprising 104 in the experimental group and 83 in the control group. The meta-analysis results are shown in Fig. 7. Compared with the control group, exercise intervention therapy improved the scores of all dimensions and the overall quality of life in AIS patients [SMD = 1.35, 95% CI (0.72, 1.98), *P* = 0.008].

### Publication Bias and Sensitivity Analysis

#### Publication Bias Analysis

**Fig. 8:**
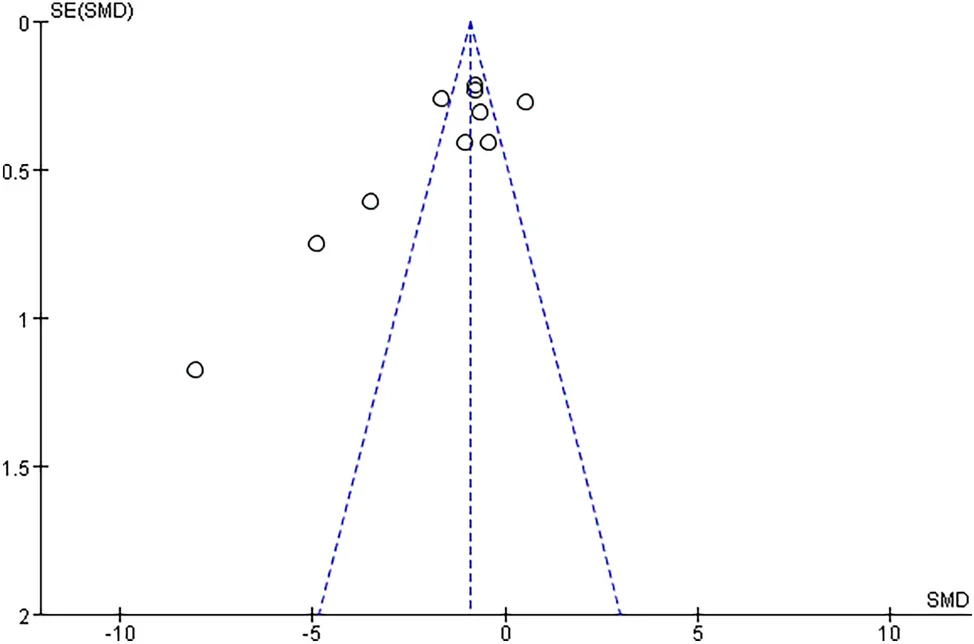
Publication bias funnel plot for the effectiveness of exercise intervention on adolescent AIS Cobb angle. Horizontal axis (X-axis): standardized mean difference (SMD), representing the effect size of each study. Vertical axis (Y-axis): standard error of SMD (SE(SMD)), reflecting the precision of each study’s effect size (smaller SE indicates higher precision). Open circle: each individual study included in the meta-analysis. The position of the circle corresponds to the study’s SMD (horizontal) and SE (SMD) (vertical). Dashed funnel shape: the theoretical range within which studies should fall if there is no publication bias. It is centered at the pooled SMD, and the width expands with increasing SE (i.e., decreasing precision). Interpretation: this funnel plot is used to assess publication bias. Asymmetry (e.g., studies missing from the lower-precision, smaller-effect side of the funnel) may indicate that smaller studies with non-significant or negative results are underrepresented, suggesting potential publication bias

The traditional funnel plot analysis examining the effectiveness of exercise interventions in improving Cobb angles among adolescent idiopathic scoliosis (AIS) patients revealed that the data from the 10 included studies did not exhibit a symmetrical distribution pattern. This indicates the presence of publication bias in the included literature, potentially attributable to small sample sizes and a wide age range among participants. Consequently, caution is warranted in interpreting the findings, as shown in Fig. 8.

#### Sensitivity Analysis of Literature

**Fig. 9:**
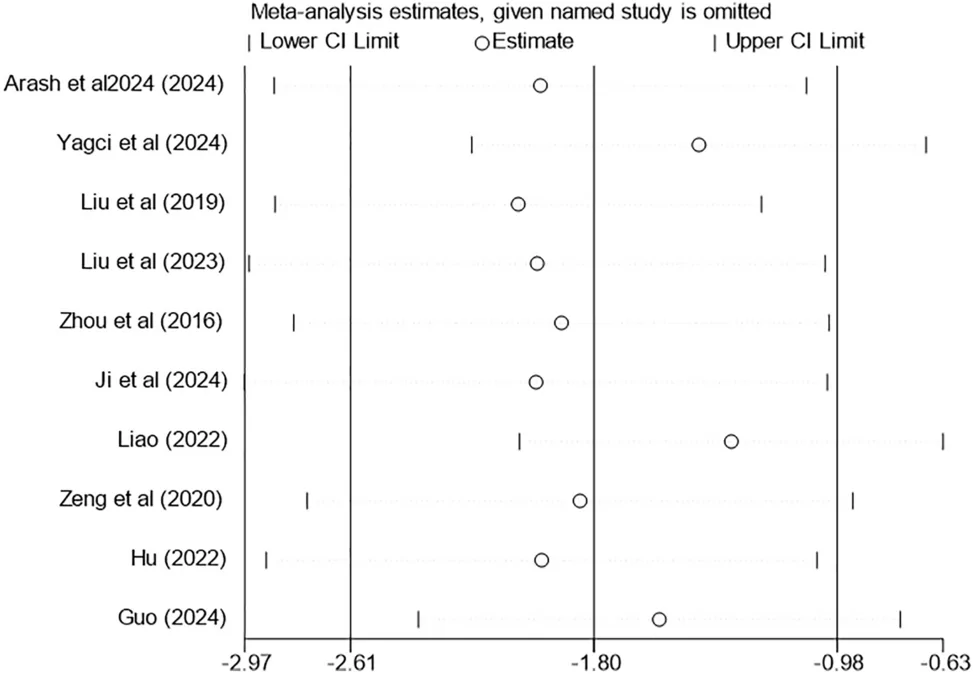
Sensitivity analysis of exercise intervention effects on adolescent AIS Cobb angle. Horizontal axis (*X*-axis): range of the 95% confidence interval (CI) for the pooled effect size. It displays the lower and upper bounds of the effect size estimate when each named study is omitted. Vertical axis (*Y*-axis): individual studies included in the meta-analysis. Each row corresponds to the scenario where the listed study is excluded. Open circle (○): the estimated pooled effect size after omitting the corresponding study, representing how the meta-analytic result changes when that specific study is removed. Vertical lines (|): the lower CI limit and upper CI limit of the pooled effect size after omitting the corresponding study, illustrating the variability of the effect size estimate in each exclusion scenario. Interpretation: this sensitivity analysis plot evaluates the robustness of the meta-analysis results. By excluding each study one by one, it determines whether any single study unduly influences the overall pooled effect. If the 95% CI of the pooled effect remains directionally consistent (e.g., does not cross zero) across all exclusion scenarios, the results are considered robust. This plot helps identify influential studies and assess the stability of the overall conclusion

To assess the robustness of the meta-analysis results, a systematic sensitivity analysis was conducted, including the sequential exclusion of each individual study. The results of this analysis are shown in the figure: after excluding any single study, the pooled effect size ranged from − 2.97 to − 0.63. Overall, the sensitivity analysis results of this meta-analysis were robust, as illustrated in Fig. 9.

## Discussion

Adolescent idiopathic scoliosis (AIS) is a three-dimensional spinal deformity with an etiology that has not been fully elucidated. Its onset is insidious, and its progression is uncertain. One of the core pathological bases of AIS is the imbalance in muscle strength on both sides of the spine. The muscles on the convex side remain in a state of long-term tension, while those on the concave side are weak, leading to a continuous displacement of the spine in the coronal plane. Exercise intervention therapy aims to break this imbalance through targeted training, which elongates the contracted tissues on the concave side, activates the weak muscle groups, and adjusts the spinal alignment to reduce the scoliosis angle. In this meta-analysis, a total of ten randomized controlled trials (RCTs) were included. The study found that exercise intervention therapy can effectively improve the effect sizes of Cobb angle, ATR, AVR, and SRS-22 scores.

In terms of the Cobb angle, the results of this study demonstrate that compared to conventional interventions, exercise intervention therapy can significantly reduce the average Cobb angle in AIS patients by 4.89°. This finding is consistent with the conclusions of multiple previous studies. For instance, the randomized controlled trial conducted by Ko et al. [28] confirmed that a 12-week core stability training program could significantly reduce the Cobb angle in adolescents with idiopathic scoliosis. Similarly, Kuru et al. [29] study on the Schroth method revealed that three-dimensional corrective training could effectively improve coronal plane spinal deformities, with a reduction in Cobb angle during the 6-month follow-up that aligned with the trend observed in our study. Furthermore, evidence compiled by Negrini et al. [30] in the SOSORT guidelines indicates that standardized exercise interventions are more effective than simple observation in controlling the Cobb angle in patients with mild to moderate AIS, further supporting the conclusions of our study. From a mechanistic perspective, the improvement in Cobb angle stems from the synergistic effects of exercise intervention therapy on spinal biomechanics and neuromuscular control. One of the core pathological bases of AIS is the imbalance in muscle strength on both sides of the spine—where the convex side muscles remain in a state of long-term tension while the concave side muscles are weak, leading to continuous coronal plane displacement of the spine [4, 31]. Exercise intervention therapy disrupts this imbalance through targeted training. Specifically, rotational breathing techniques elongate the contracted tissues on the concave side, activate the weak muscle groups, and adjust spinal alignment to reduce the scoliosis angle [18, 29]. Core stability training enhances the coordinated contraction of the lumbar, abdominal, and paraspinal core muscle groups, providing a more stable support framework for the spine and delaying or reversing scoliosis progression [21, 28]. The SEAS (Scientific Exercises Approach to Scoliosis) therapy emphasizes active self-correction and functional integration, reducing the adverse effects of abnormal stress on spinal growth by repeatedly reinforcing neuromuscular memory in correct postures [19, 32]. These mechanisms work together to ultimately result in a significant reduction in the Cobb angle, confirming the core value of exercise intervention therapy in correcting coronal plane spinal deformities.

In terms of the angle of trunk rotation (ATR), the results of this study reveal that exercise intervention therapy can significantly reduce the average ATR in adolescent patients with AIS by 1.80°, a finding consistent with the conclusions of multiple studies. Specifically, Hu Lijie et al. [23], in their study on the Barcelona Scoliosis Physical Therapy School (BSPTS) method, found that a 6-month program of specific exercise training could reduce the average ATR by 2.1°. The mechanism underlying this effect may be related to the method's use of three-dimensional correction techniques to adjust the spatial position of the trunk and activate the deep rotational muscle groups of the trunk. Similarly, Yagci et al. [19], in a 1-year intervention study using the Scientific Exercises Approach to Scoliosis (SEAS) method, demonstrated that this therapy, through active self-correction training to enhance neuromuscular control, could reduce the ATR by 1.8°compared to the control group, with this improvement maintained during long-term follow-up. From a biomechanical perspective, the improvement in ATR stems from the targeted adjustment of rotational imbalances in the spine-pelvis complex by exercise intervention therapy. Patients with AIS often exhibit concurrent abnormalities in trunk rotation and pelvic tilt. Exercise interventions, such as rotational breathing training and core stability exercises, can effectively activate rotational muscle groups, including the obliques and latissimus dorsi, thereby correcting trunk displacement by enhancing bilateral muscle coordination [33]. Recent research further supports the effectiveness of exercise intervention therapy in improving ATR. A study supported by the Scoliosis Research Society (SRS) showed that a comprehensive intervention program combining multiple exercise therapies could reduce the average ATR by 2.5°over a 9-month intervention period, with significant improvements in both functional and quality of life scores among patients [34]. These interventions work synergistically to achieve a significant reduction in ATR, confirming the important value of exercise intervention therapy in improving horizontal plane spinal deformities.

The pathological characteristics of AIS encompass not only abnormal Cobb angles in the coronal plane and altered physiological curvature in the sagittal plane, but also vertebral rotation in the horizontal plane. AVR serves as the core metric for quantifying this horizontal plane deformity, and it is closely correlated with the progression of scoliosis, trunk deformity and treatment difficulty. It represents a crucial dimension for assessing the severity of AIS and evaluating treatment outcomes. Current research remains inconclusive regarding the efficacy of exercise interventions in improving AVR. Some studies indicate specific exercise therapies can significantly reduce AVR. Mohamed et al. [35] compared the Schroth three-dimensional therapy with proprioceptive neuromuscular facilitation techniques, finding that Schroth therapy adjusted vertebral spatial positioning through rotational breathing exercises, resulting in an average AVR reduction of 2.3°. However, our analysis revealed only a mean reduction of 0.13°in AVR, indicating that exercise interventions do not significantly improve AVR.This discrepancy primarily stems from three factors. First, limitations in the number of included studies and sample sizes. This review incorporated only threeAVR-related studies with small sample sizes, and some positive-result studies had even smaller samples, potentially leading to insufficient statistical power. Second, variations in intervention duration and precision. As a rotational indicator of deep vertebral structures, AVR improvement requires long-term, mechanism-specific training. Some interventions in this review were short-term, potentially failing to yield significant effects. Third, differences in measurement sensitivity. Variations in the consistency and sensitivity of AVR assessment tools across studies may introduce bias. Future high-quality research is needed to further validate the impact of exercise on AVR.

Patients with adolescent idiopathic scoliosis (AIS) often experience psychological issues such as self-image distress and social avoidance due to the abnormal physical appearance caused by spinal deformities. Simultaneously, restricted spinal function may impact daily activities, triggering pain or fatigue, thereby reducing their quality of life. The Scoliosis Research Society-22 (SRS-22) questionnaire, developed by the Scoliosis Research Society, provides a comprehensive evaluation across five dimensions: pain, functional activity, mental health, self-image, and treatment satisfaction. It reveals that AIS patients generally score lower than healthy adolescents in both the total score and each dimension, with particularly pronounced impairments in self-image and mental health. Exercise interventions have demonstrated a clear positive effect on improving the quality of life of AIS patients. The results of our meta-analysis show that exercise interventions can significantly increase the SRS-22 score by 0.53 points, with notable improvements in the mental health and functional activity dimensions. This finding is consistent with the conclusions of multiple previous studies. Khaledi et al. [18] conducted a randomized controlled trial demonstrating that a 12-week Schroth therapy program could increase the total SRS-22 score by 26% in AIS patients, with the most significant improvement observed in the self-image dimension. Hu Lijie [23] pointed out in their study that the group training model in specific exercise therapies can promote mutual support among patients, further enhancing treatment confidence and improving scores in the treatment satisfaction dimension. Yagci et al. [19], in their study on the Scientific Exercises Approach to Scoliosis (SEAS) method, also found that after one year of intervention, patients' scores in mental health and treatment satisfaction increased by 1.2–1.5 points compared to the control group, confirming the multidimensional improvement effect of exercise on quality of life. Research indicates that exercise intervention therapies can enhance quality of life through a dual "physiological–psychological" pathway. At the physiological level, improvements in spinal deformities directly alleviate physical discomfort and enhance motor ability, enabling patients to more easily participate in daily activities and social interactions, thereby improving functional activity scores. At the psychological level, active control and correction of posture during exercise training can enhance patients' sense of control over their bodies, reducing feelings of inferiority caused by abnormal appearance [29, 36]. Additionally, the release of endorphins during regular exercise can alleviate anxiety and depressive tendencies, improving mental health status [37].

Limitations and shortcomings of this study: (1) among the ten included studies, some exhibited deficiencies in aspects such as random sequence generation and blinding implementation, which may have introduced bias; (2) The varying degrees of scoliosis and different intensities of exercise interventions included in the studies could potentially lead to result bias; (3) The limited number of studies incorporating metrics like axial vertebral rotation (AVR) resulted in insufficient result stability; (4) only Chinese and English literature were included, which may introduce language bias.

In summary, this study, through meta-analysis, confirmed the effectiveness of exercise intervention therapy in improving Cobb angle, angle of trunk rotation (ATR), and quality of life in patients with adolescent idiopathic scoliosis (AIS). However, due to limitations such as the number of included studies, intervention duration, and measurement methods, no significant effect was observed in improving AVR. Future research should focus on conducting more high-quality, large-sample, multicenter studies to further explore the therapeutic effects and mechanisms of exercise intervention therapy for AIS.

## Conclusion

To sum up, exercise intervention therapy yields significant beneficial radiological and clinical effects in adolescents with AIS. It significantly improves Cobb angle and ATR and enhances quality of life (SRS-22 scores), whereas no significant improvement is observed in apical vertebral rotation (AVR). Despite the certain risk of bias in the existing included studies and the small sample size related to AVR, exercise intervention therapy can still provide a feasible core option for non-surgical conservative treatment of AIS patients, especially for those with mild to moderate scoliosis. It is reasonable for future studies to emphasize the conduct of high-quality, multicenter randomized controlled trials (RCTs) to verify its long-term efficacy and optimize exercise protocols targeting AVR.

## Data Availability

All data generated or analyzed during this study are included in this published article (and its Supplementary Information files).

## Abbreviations

AIS: Adolescent idiopathic scoliosis
ATR: Angle of trunk rotation
AVR: Apical vertebral rotation
RCT: Randomized controlled trial
SMD: Standardized mean difference
BSPTS: Barcelona School of Physical Therapy Scoliosis Method
SEAS: Scientific Exercises Approach to Scoliosis
SRS-22: Scoliosis Research Society-22
